# Maternal and neonatal outcomes of intrahepatic cholestasis of pregnancy after in vitro fertilization

**DOI:** 10.1186/s12884-024-06248-x

**Published:** 2024-01-08

**Authors:** Ying Zhu, Le Xu, Rajluxmee Beejadhursing, Fei Li

**Affiliations:** 1 Department of Obstetrics and Gynecology, Tongji Hospital, Tongji Medical College, Huazhong University of Science and Technology, Wuhan, China; 2 Reproductive Medicine Center, Tongji Hospital, Tongji Medical College, Huazhong University of Science and Technology, Wuhan, China

**Keywords:** Intrahepatic cholestasis of pregnancy, In vitro fertilization, Maternal complications, Neonatal outcomes

## Abstract

**Background:**

Intrahepatic cholestasis of pregnancy (ICP) is an idiopathic disease of pregnancy. Little is known about how it specifically affects pregnancies resulting from in vitro fertilization (IVF). Our aim is to evaluate the impact of IVF on the perinatal outcomes of ICP.

**Methods:**

A retrospective study of 242 patients with intrahepatic cholestasis of pregnancy, comprising 36 conceived through IVF and 206 spontaneous conceptions (SC), enrolled between 2019 and 2021 was carried out. Data were analyzed from the medical archives of the Huazhong University of Science and Technology, Tongji Hospital.

**Results:**

Numerical values of transaminases (ALT, alanine aminotransferase; AST, aspartate aminotransferase) and serum total bile acid (TBA) are significantly lower in the IVF group than that in the spontaneous conceived group (*p* < 0.05). The incidence of gestational diabetes mellitus (GDM) was higher in the IVF group than in SC group (30.6% vs. 16%, *p* = 0.037). The cesarean section (CS) rates are higher in the IVF group (97.2% vs. 85.4%, *p* = 0.023). On the other hand, the prevalence of premature rupture of membranes (PROM) was higher in the SC group (10.7%) while none was reported in the IVF-ICP group. Other maternal comorbidities and neonatal outcomes were similar between the two groups.

**Conclusion:**

ICP patients who underwent IVF are more likely to suffer from GDM. Therefore, monitoring and management of blood glucose should be strengthened during pregnancy. Fortunately, IVF does not seem to worsen the progression or outlook of ICP, so sticking to standard management practices is recommended.

## Introduction

Intrahepatic cholestasis of pregnancy is an idiopathic pregnancy-related condition characterized by pruritus and elevated concentrations of total bile acids. Patients typically recover after delivery [1]. The incidence rates vary between 0.3 and 5.6% of pregnancies [2]. ICP is associated with a higher incidence of adverse perinatal outcomes in the third trimester, such as spontaneous or iatrogenic premature delivery, meconium staining of the amniotic fluid, foetal distress, neonatal asphyxia and even stillbirth [3]. Women with ICP also have a higher incidence of premature rupture of membranes, postpartum haemorrhage, as well as increased risk of gestational diabetes and hypertension [4, 5].

The pathogenesis of ICP is still not completely understood, though, gestational hormones, environmental factors, genetic variation and lipid metabolism contribute to the development of this disease [6, 7]. Current evidence suggest that assisted reproductive technology (ART) may promote the occurrence and development of ICP [8–10]. A significant proportion of women conceiving through assisted reproduction treatments have hormonal dysfunction and multiple metabolic abnormalities, contributing to obstetric complications [11, 12]. Despite the increasing number of births from assisted reproductive technology, knowledge regarding ICP outcomes after IVF remains limited, particularly the specific maternal characteristics and perinatal outcomes. The aim of this retrospective cohort study is to explore the impact of IVF on perinatal outcomes associated with ICP and to determine the optimal management model for IVF-related ICP patients.

## Materials and methods

### General Information

This study is a retrospective analysis of data from 242 pregnant women diagnosed with ICP, including 36 who conceived through IVF and 206 spontaneous conceptions. All patients delivered between January 2019 and December 2021 at the Tongji Hospital of Tongji Medical College of Huazhong University of Science and Technology, Wuhan, China.

### Diagnostic criteria

ICP was defined as manifestation of pruritus in the absence of rash, together with raised level of serum bile acids (cut-off level 10 µmol/L) and/or raised level of serum ALT (> 40 U/L), and by normalization of biochemical parameters after delivery [13].

Gestational diabetes mellitus was established following the “one-step” 75-g oral glucose tolerance test [14].

### Exclusion Criteria

Incomplete case data, twin pregnancy, multiple pregnancy, extrahepatic biliary tract obstructions, viral hepatitis, autoimmune hepatitis, HELLP syndrome and fatty liver of pregnancy.

### Statistical Processing

Statistical analysis was performed using SPSS 20.0 statistical software. Continuous data are presented as mean and standard deviation, while categorical data as number and percentage. The significance of the difference between the mean values of the groups was evaluated using the Student’s t-test and the significance of the difference in the median values was evaluated using the Mann-Whitney U test. Categorical data was compared by the chi-square distribution. The odds ratios (OR) and corresponding 95% confidence intervals (CI) for the risk of GDM were calculated. A p-value of < 0.05 was considered statistically significant.

## Results

**Fig. 1:**
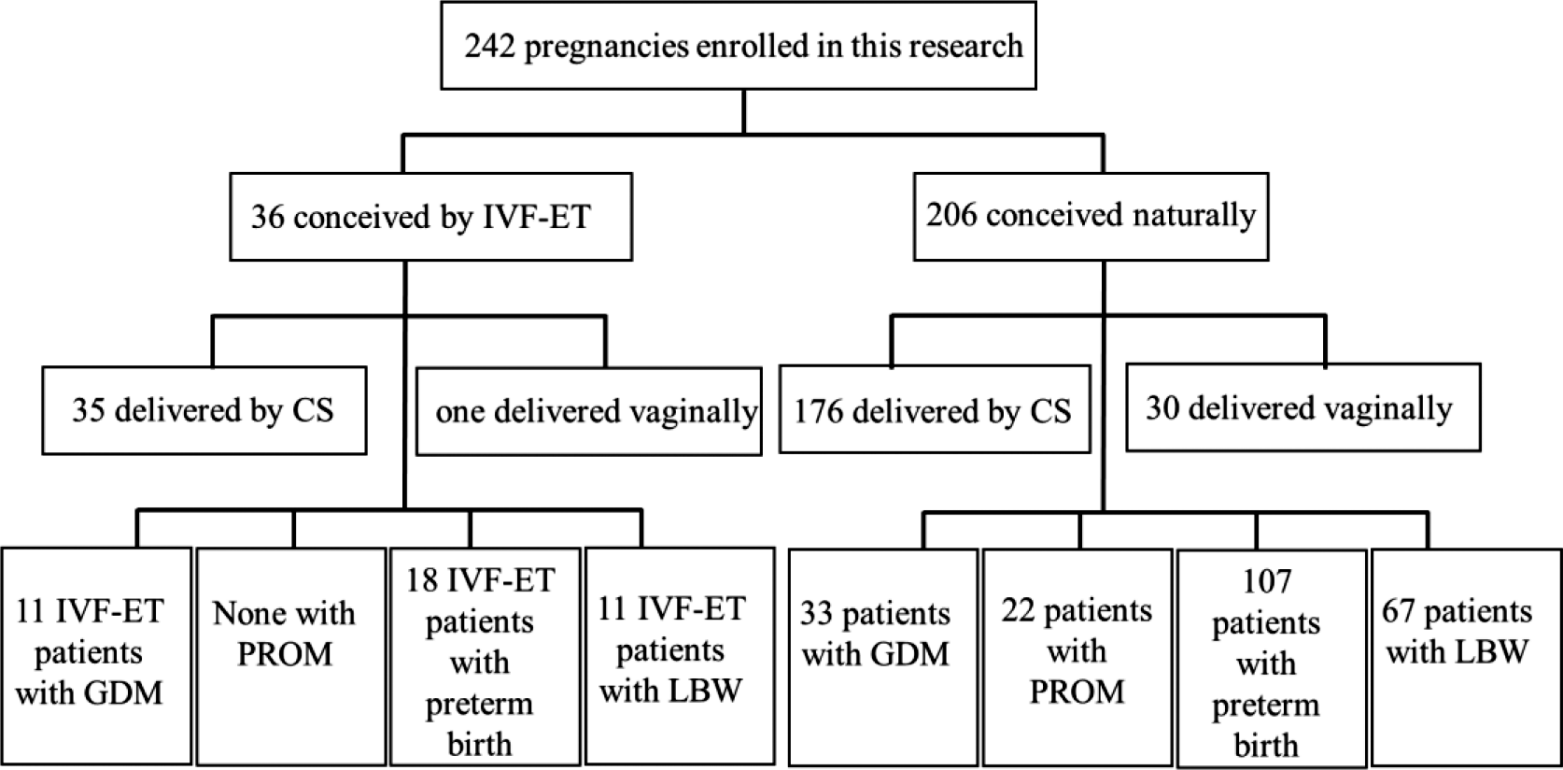
Flowchart of participants in the analysis. LBW: Low birth weight

As Table 1 illustrates, there was no significant difference in age, pregnancy or delivery histories between the two groups (*p* > 0.05). The mean gestational age at onset of ICP was 35.5 weeks for both groups. Figure 1 shows the flow chart of the participants in the main analysis.

**Table 1:** Maternal characteristics comparison between the SC and IVF groups

Characteristics	SC group (*n* = 206)	IVF group (*n* = 36)	P-Value
Age (years)	29.6 ± 4.5	31. 9 ± 3.8	0.286
Onset time of symptoms (weeks)	35.5 ± 3.1	35.5 ± 4.7	0.414
Gestational age at delivery (weeks)	36.2 ± 2.5	36.7 ± 2.2	0.358
primipara	100	30	-
multigravida	106	6	-
Serum ALT(IU/L)	139.3 ± 192.6	55.9 ± 95.8	0.01
Serum AST(IU/L)	112.3 ± 161.3	57.4 ± 87.9	0.025
TBA (µmol/L)	44.8 ± 35.7	29.1 ± 23.1	0.005

Woman in the IVF pregnancy cohorts exhibited higher rates of GDM compared to the SC group (30.6% vs. 16%, *p* = 0.037). In addition, IVF group also had a notably higher cesarean section rates (97.2% vs. 85.4%, *p* = 0.023).

Conversely, premature rupture of membranes was more prevalent in the SC group (10.7%) while no such occurrence was reported in the IVF-ICP group. Meanwhile, the levels of transaminases (ALT, AST) and TBA are greatly reduced in the IVF group than that in the SC group (*p* < 0.05) (Table 1).

The severity of ICP, defined as TBA exceeding 100 µmol/L, was 2.8% in the IVF group compared to 9.2% in the SC group. There were no significant differences observed between the two groups in terms of other maternal complications and neonatal outcomes, such as postpartum haemorrhage, gestational hypertension, and preterm labor (Table 2).

**Table 2:** Analysis of maternal complications and neonatal outcomes

Items	SC group (*n* = 206)	IVF group (*n* = 36)	P-Value
Gestational hypertension	26	8	0.127
GDM	33 (16.0%)	11 (30.6%)	0.037
PROM	22	0	0.040
Postpartum hemorrhage	12	2	0.949
Vaginal delivery	30	1	0.015 0.023
Cesarean section	176	35	
Preterm birth	107	18	0.830
Meconium-stained amniotic fluid	47	6	0.531
Stillbirth	0	0	-
Asphyxia neonatorum	48	4	0.816
Low birth weight	67	11	0.816

## Discussion

Interestingly, this study demonstrated that even though ICP incidence is higher, TBA, AST, and ALT mean values are significantly lower in ICP pregnancies resulting from IVF. This difference may be attribute to the benefit of more intensive prenatal care and focused treatment provided to IVF patients. Remarkably, only 1 out of 36 patients of the IVF cohort reported a TBA that surpassed 100 µmol/L. Certain studies have indicated that concentration of serum bile acid correlates to perinatal complications. Once TBA exceeds 40 µmol/L, for each 1 µmol/L increase, the incidence of perinatal complications will go up by 1–2%. If it reaches 100 µmol/L, the risk of stillbirth elevates by 30 times [15, 16]. Although assisted reproduction might induce ICP, no significantly increase in adverse outcomes were detected in this data set. No obvious difference between the two groups in incidence of pre-preeclampsia, postpartum haemorrhage and coagulation disorders was demonstrated. Nevertheless, IVF patients showed a higher likelihood of developing gestational diabetes. In this study, pregnancies conceived by IVF had a higher incidence rate of GDM, OR 1.91 (95% CI 1.06–3.42). Infertility secondary to ovulatory disorders often present with serious endocrinological disorders, such as insulin resistance, or high risk factors for obstetric diseases, for instance, obesity [17]. Excessive progesterone could interfere with the function of major hepatic bile acid receptors, potentially exacerbating the situation in ICP patients [18]. Experimental report indicate that oestrogen could trigger glycemic disorders resulting in liver injury and exacerbating cholestasis [19]. For ICP in patients having undergone IVF, careful attention to gestational glucose status is crucial for early management, potentially alleviating later pregnancy burdens.

ICP is a polygenic disorder, and its specific pathogenesis has not been fully understood. Recent literature report that cholestasis may also be related to mutations in genes encoding bile acid metabolic transporters such as ABCB11, ABCB4, ABCCC2, ATP 8B1, TJP2, which result in the abnormal function of bile salt export pump in hepatocytes [20, 21]. Hormonal imbalances induced by assisted reproductive technology, particularly high oestrogen and progesterone levels, are more likely to contribute to ICP, and lead to adverse pregnancy outcomes [22, 23]. Redundant oestrogen accumulates in liver cells, leading to decreased activity of Na^+^/K^+^-ATPase, which can inhibit the synthesis of hepatic biliary proteins, and obstruct of bile salt uptake and excretion [24]. Supplementary progestin administration in IVF patients could trigger bile acid retention, thereby causing adverse events, such as premature birth, respiratory disorders, meconium-stained amniotic fluid and even stillbirth [13]. Serum bile acids stimulate the release of prostaglandins from the uterus and decidua, increase the expression of oxytocin receptors in uterine smooth muscle, and induce preterm delivery [25, 26]. High bile acid concentrations may cause hypoxia which can stimulate foetal vagus nerve, increase intestinal peristalsis, and result in intrauterine meconium passage. As the fetus inhales the tainted amniotic fluid, excessive bile acid accumulation can result in atelectasis, pulmonary edema, neonatal lung injury or even asphyxia [27, 28].

The most tragic outcome of ICP is stillbirth whose exact mechanism has yet to be fully clarified. Fetal cardiomyocytes are more susceptible to bile acids compared to adults. Once bile acids enter systemic circulation, they can directly impair fetal cardiac conduction system, reduce cardiac systolic function and induce fetal arrhythmia [29–31]. Additionally, it has also been suggested that bile acids stimulate blood vessels on the chorionic surface of the placenta, causing vasospasm and decreasing placental perfusion, leading to sudden death [32]. Studies report an incidence of stillbirth of 18.3% at 36 weeks, escalating to 33.6% at 39 weeks [33]. Hence, inclusion of all members of the multidisciplinary team involved in the process is recommended, namely, gynecologists, midwives, neonatologists, pediatricians, and physicians. This collaborative approach will definitely reassure and comfort patients that a team is dedicated to everyone’s safety.

Prompt and effective management of severe ICP significantly reduces the risk of adverse perinatal outcomes [34]. For IVF patients with ICP in this study, operative delivery was personally preferred to minimize emergence of adverse perinatal events, reflected as a high rate (97.2%) of cesarean section. However, prenatal monitoring and the timing of delivery must be a comprehensive consideration [23]. Termination of pregnancy around 36 weeks did not increase the incidence of stillbirth in this study. Extending gestational age could reduce the incidence of iatrogenic premature birth. Delivery timing should not only depend on the concentrations of serum total bile acid, but also on feedback from prenatal monitoring such as Non-Stress Test, fetal growth, gestational age and the effectiveness of medical treatment.

This study has analyzed IVF as a modern effective way of achieving pregnancy that does not increase adverse complications in ICP. Therefore, it is advisable to continue routine prenatal check-up. These findings may aid in optimizing medical resources and alleviating emotional tension relating to infertility. Nonetheless, there are two limitations that require improvement. Firstly, the sample size was limited without post-hoc power analysis, an assessment of neonatal prognosis or quality of life. Future studies should aim to include larger samples and provide comparative analyses. The results would help in identifying individuals at risk, particularly with regards to mixed-etiologies, early diagnosis, prevention and possible targeted treatment technologies. Besides, optimal delivery timing for patients with ICP need to be addressed to reduce the burden of prematurity. Secondly, there were no cases of stillbirth, necessitating further research in its etiology and prevention. Despite these limitations, the data presented in this study provides a general evaluation of the impact of assisted reproduction on patients with ICP.

## Conclusion

Through this retrospective, hospital-based cohort study, we discovered that ICP patients who underwent IVF did not demonstrate a heightened risk of preterm birth, gestational hypertension, and postpartum haemorrhage compared to cohorts who conceived spontaneously. However, IVF-related ICP may increase incidence of glucose metabolism disorders, artificial reasons-caused prematurity, and cesarean sections. Therefore, it is advisable for women with ICP in post-IVF pregnancies to follow medical advice for proper monitoring and management. This approach guarantees necessary interventions, prevent complications and reduces unnecessary cesarean sections.

## Data Availability

The datasets used and/or analyzed during the current study will be available from the corresponding author on reasonable request.

## Abbreviations

ICP: Intrahepatic cholestasis of pregnancy
IVF: In vitro fertilization
CS: cesarean section
TBA: Serum total bile acid
ALT: Alanine aminotransferase
AST: Aspartate aminotransferase
SC: Spontaneous conceived
GDM: Gestational diabetes mellitus
ART: Assisted reproductive technology
OR: Odds ratio
CI: Confidence interval
PROM: Premature rupture of membrane
LBW: Low birth weight
